# Innovative Multilayered Electrospun Fiber Systems for Dual-Action HIV Prophylaxis and Nonhormonal Contraception

**DOI:** 10.1155/adpp/4749211

**Published:** 2025-06-29

**Authors:** Deborah A. Ogundemuren, Peace-Ofonabasi O. Bassey, Karamot O. Oyediran, Ohakwe G. Nmesoma, Dimitrios Tsamos, Alkiviadis Tsamis, Alexander E. Tsouknidas, Andrew N. Amenaghawon, Chukwuemeka P. Azubuike, Margaret O. Ilomuanya

**Affiliations:** ^1^Department of Pharmaceutics and Pharmaceutical Technology, Faculty of Pharmacy, University of Lagos, PMB 12003, Lagos, Nigeria; ^2^Department of Mechanical Engineering, School of Engineering, University of Western Macedonia, Kozani 50100, Greece; ^3^School of Engineering, University of Leicester, Leicester LE1 7RH, UK; ^4^Department of Chemical Engineering, Faculty of Engineering, University of Benin, Benin City, Nigeria

**Keywords:** CatSper channel blocker, electrospun fibers, multipurpose prevention technology, pH-responsive drug release, tenofovir

## Abstract

**Background:** Electrospun fiber drug delivery systems, integrated into multipurpose prevention technologies, offer a promising solution for women facing health risks from HIV/STIs and unmet contraceptive needs by providing on-demand protection in a single dosage form. This study investigates the potential of a multilayer electrospun fiber construct for pH-responsive and sustained release of the HIV microbicide tenofovir (TFV) and the CatSper channel blocker nifedipine (NFP) respectively.

**Method:** Electrospun fibers were fabricated in a stacked architecture by blend electrospinning using polycaprolactone (PCL) as the backing layer for delivering NFP and cellulose acetate phthalate (CAP) as the top layer for delivering TFV. An analysis of surface morphology, mechanical and chemical properties, mucoadhesion, drug release profiles, encapsulation efficiency, and safety assessments was performed.

**Results:** An encapsulation efficiency of 52.13% was achieved for TFV, with a drug loading of 7.00%, while for NFP, the encapsulation efficiency was 63.86%, with a drug loading of 0.56%. The top layer exhibited a pH-responsive release profile and Fickian diffusion in both SVF and SVF/SF environments, while the backing layer showed Fickian diffusion in SVF and a release profile closer to zero-order in SVF/SF.

**Conclusion:** This study highlights the potential of multilayered CAP/PCL electrospun fibers for intravaginal delivery of TFV and NFP, aimed at the pre-exposure prophylaxis of HIV-1 and prevention of unplanned pregnancy.

## 1. Introduction

The syndemics of human immunodeficiency virus (HIV)/sexually transmitted infections (STIs) and the persisting lack of access to modern contraceptive methods continue to pose substantial challenges to women's health and well-being globally. [1]. In 2022, women and girls of all age groups constituted 46% of all new HIV infections globally. Within Sub-Saharan Africa, adolescent girls and young women accounted for more than 77% of new infections among those aged 15–24 years [2].

From 2015 to 2019, an estimated 121 million unintended pregnancies occurred globally each year, corresponding to a worldwide rate of 64 unintended pregnancies per 1000 women aged 15–49 years [3].Studies in low- and middle-income countries have shown consistently low utilization of long-acting modern contraceptive methods [4]. Many women still distrust hormonal birth control due to concerns about drug interactions, obesity, and impacts on fertility. Therefore, nonhormonal methods that are easily accessible and do not get absorbed into the body are often preferred [5].

Multipurpose prevention technologies (MPTs) offer a potential solution for meeting a range of sexual and reproductive health (SRH) needs. These technologies aim to prevent unintended pregnancies, and other STIs, particularly among vulnerable groups such as adolescent girls and young women [6].

Several attempts have been made to create nonhormonal MPTs for vaginal application. For instance, Weitzel et al. created a nonhormonal MPT vaginal gel using polyphenylene carboxymethylene (PPCM), which has demonstrated activity against HIV-1, herpes simplex virus (HSV-1 and HSV-2), as well as acting as a noncytotoxic contraceptive [5]. Another innovative nonhormonal topical nanosystem was developed by Ball et al., which contains drug-loaded nanofiber (NF) meshes targeting HIV and sperm motility [7].

Electrospun NFs have emerged as highly promising in drug delivery systems due to their exceptional characteristics. They possess a high surface-to-volume ratio, small dimensions, and are highly adjustable. These qualities enable NFs to carry drugs efficiently, offering substantial drug loading capacity, precise control over release profiles, and improved stability [8]. Furthermore, as a solid dosage form, NFs address challenges associated with liquid or gel-based formulations, such as messiness and leakage [9]. In a recent exploratory study, women indicated a preference for fiber-based mat-type dosage forms over gels and films, finding them more convenient [10]. Kim Woodrow et al. presented various fiber designs for the co-delivery of multiple medications to prevent sexual transmission of HIV-1/HSV-2 and for contraception [11].

Cellulose acetate phthalate (CAP) fibers have been investigated for their potential in HIV prevention, with studies, such as that of Huang et al., demonstrating the successful incorporation of antiretroviral drugs such as tenofovir (TFV) disoproxil fumarate (Viread) into CAP-based delivery systems. These findings highlight the suitability of CAP fibers for localized delivery of HIV prophylactic agents. In the present study, we introduce a distinct strategy by developing a multilayer fiber system designed as a MPT. This system comprises two separate layers—, one formulated for HIV prevention and the other for nonhormonal contraception, with the goal of offering simultaneous protection against both HIV infection and unintended pregnancy in a single platform.

Therefore, we developed pH-responsive CAP and polycaprolactone (PCL) multilayer electrospun fibers for the on-demand release of TFV and sustained nifedipine (NFP) release. This safely and effectively inhibits both the HIV and spermatozoa in the vaginal environment. TFV, an adenosine nucleotide analog, exhibits activity against HIV and HSV-2 and has been demonstrated to reduce the acquisition of HIV [12]. NFP is a non-specific L-type calcium channel blocker that has been shown to inhibit sperm motility thereby serving as an on-demand contraceptive [13].

## 2. Materials and Methods

### 2.1. Materials

These included PCL (MW: 50,000–80,000; CAS no. 24980-41-4), TFV (Energy Chemical Inc., Shanghai), polyethylene glycol (PEG) (MW: 2000), CAP (Shanghai Macklin Biochemical Co., China), acetone and chloroform (CAS No. 67-66-3) (Molychem., India), and ethanol (CAS No 64-17-5) (Honeywell., Germany). Porcine stomach mucin-type II (CAS no. 84082-64-4), NFP tablets (Nifedin Dexcel 20 Retard NAFDAC no: 04-4499 Batch no: 2284199), and deionized water were obtained from the Central Research Laboratory, LUTH. Simulated vaginal fluid (SVF) was prepared as described in [14]. The reagents used were of high purity and were utilized as received, with no additional purification.

### 2.2. Engineering of Drug-Loaded CAP/PCL Electrospun Fibers

TFV and NFP-loaded CAP/PCL multilayered fibers were produced using multineedle blend electrospinning. Initially, 12% and 17% CAP solutions were prepared in acetone: ethanol (1:1) mixture at room temperature and stirred for 3 h to ensure an even distribution of components. A 17% w/v CAP solution was found to have a suitable viscosity for spinning NFs. Next, 10%w/w PEG was added to the CAP solution and stirred for 2 h. TFV-loaded NFs were then created by dissolving 1 g of TFV in 100 mL of the polymer mixture to achieve a theoretical drug loading of 1% w/v TFV.

A 15% w/v PCL polymer solution was prepared as the backing layer by dissolving PCL beads in a 1:1 (v/v) chloroform and ethanol mixture at room temperature, allowing the solution to mix overnight. The NFP-loaded polymer mixture, with a theoretical drug loading of 0.05% w/v NFP, was made by adding 50 mg of NFP to 100 mL of the 15% w/v PCL solution (Table 1).

#### 2.2.1. Electrospinning Process

Electrospun fibers were produced from polymeric solutions utilizing the NLI® electrospinning machine (Model BS-35CL-2-ESD). The electrospinning setup included a high-voltage power source, a syringe pump with five plastic syringes, flat-tip needles, and a grounded rotating drum collector. The process involved injecting the polymer solution into the plastic syringes, which were fitted with metal needle tips. A multineedle array was used, with all syringes mounted on the pump, and the voltage was applied through the alligator clip. Activation of the high-voltage supply created an electric field between the needles and the rotating collector, facilitating the electrospinning process. The electrospinning process for the multilayered NFs began with the first layer, where 2.5 mL of PCL solution was spun from each syringe fitted with an 18 G needle tip. For the second layer, 5 mL of CAP/PEG solution was spun from each syringe equipped with a 21 G needle tip. The voltage applied was 15 kV for the CAP/PEG solution and 23 kV for the PCL solution. Both layers were controlled at a 1 mL/h flow rate, monitored by the syringe pump.

During spinning, fibers were collected onto an 8-cm diameter stainless steel drum rotating at 500–520 rpm, positioned 15 cm from the needle tip for the first layer (PCL) and at 30 rpm for the second layer (CAP/PEG) at the same distance. This setup promoted the formation of well-aligned fibers. The drum was covered with aluminum foil to facilitate the recovery of the final NF sheet.

After completing the electrospinning process, the aluminum foil was taken off the drum, and the NFs were carefully removed. The fibers then underwent air-drying at 25°C ± 2°C for 24 h to ensure complete evaporation of the solvent before further analysis.

### 2.3. Surface Morphology of Electrospun NFs

Scanning electron microscopy (SEM) was employed to analyze the surface structural characteristics of the NFs. Samples were placed on carbon tape, coated with a thin layer of gold, and imaged using a JEOL JSM-6610LV SEM. Fiber diameters were measured using ImageJ (National Institutes of Health [NIH], USA), with the average diameter calculated from at least 80 fibers per image. The distribution of fiber diameters was analyzed using GraphPad Prism 6 (Prism Software, USA). The porosity of the nanofibrous membranes was assessed by analyzing binary images with ImageJ software. This involved defining the fiber matrix and pores by carefully selecting appropriate upper and lower threshold values, followed by calculating the percentage of the area occupied by pores based on pixel analysis [15].

### 2.4. Mechanical Characterization

The mechanical properties of the NFs were assessed using a universal mechanical testing machine (Instron Series 3369, USA) with a 50 kN load measurement capability. Thin sections of 5 × 10 mm (length × width) were stretched using a gauge length of 50 mm, under ambient conditions of 25°C and 60% relative humidity. The maximum load and strain at break were recorded in triplicate.

### 2.5. Chemical Characterization

#### 2.5.1. X-Ray Diffraction (XRD)

The XRD spectra of the electrospun NFs were obtained with an X-ray diffractometer (Rigaku Miniflex, Japan) with Cu Kα radiation (*λ* = 1.5418 Å). The instrument was used at a fixed current (I = 15 mA) and voltage (*U* = 30 kV), and measurements were taken over a range of 2*θ* from 2° to 145°.

#### 2.5.2. Fourier Transform Infrared Spectroscopy (FTIR)

An FTIR spectrophotometer (Agilent Cary 630 ATR-FTIR) was utilized to identify specific functional groups, chemical bonds, and molecular interactions within the NFs. The spectra were measured from 4000 to 500 cm^−1^. Prior to analysis, the NFs were vacuum-dried at 45°C and positioned on a diamond crystal for measurement. To minimize noise while preserving peak intensity, smoothing was applied when required [15].

#### 2.5.3. Differential Scanning Calorimetry (DSC)

A differential scanning calorimeter (DSC 2/700/14, Mettler Toledo, USA) was employed to assess the heat flow linked to phase transitions and the physicochemical characteristics of the NFs. Samples weighing 5 mg were placed in hermetically sealed aluminum pans and analyzed over a temperature range of 37°C–450°C at a temperature increase of 10°C/min. The analysis was conducted under a nitrogen purge with a gas flow of 50 mL/min [15].

### 2.6. *In Vitro* Mucoadhesion Analysis

The mucoadhesive potential of all membranes was evaluated using the periodic acid–Schiff colorimetric assay. Standard solutions of mucin (Porcine stomach mucin-type II, Sigma-Aldrich, UK) were prepared at concentrations of 0.00156, 0.03125, 0.0625, and 0.125 mg per 100 mL of phosphate-buffered saline (PBS) (pH 7.4). To assess interactions between NFs and mucin, 10 mg of each NF sample was added to the 0.125 mg/mL mucin solution. The mixtures were incubated in a shaking water bath set to 37°C for 30 min. Following incubation, the samples were centrifuged at 4000 rpm for 2 min to separate the supernatant from the NF–mucin complex.

The supernatant was then used to measure the free mucin content. To each 2 mL supernatant sample, 200 μL of 10% periodic acid was added and the mixture was incubated for 2 h, following the method of Meng et al. [16]. Free mucin was determined by measuring absorbance at 555 nm by using UV/Vis spectrophotometry (UV-2005, J.P. Selecta, Spain). The mucin adsorption (%) was calculated using the following equation:

(1) $$\begin{array}{c} \text{mucin adsorption }\left(\%\right)=\frac{\text{total mass of mucin}-\text{free mucin}}{\text{total mass of mucin}}\times 100\%. \end{array}$$

### 2.7. Drug Loading and Entrapment Efficiency

To assess drug loading, TFV-loaded NFs were weighed and submerged in 5 mL of PBS (pH 7.4) for 2 h to allow the complete dissolution of the CAP fiber. The solution was vortexed for 5 min and analyzed using a UV spectrophotometer at 260 nm. The percentage drug loading was calculated by using the following formula:

(2) $$\begin{array}{c} \text{drug loading }\left(\%\right)=\frac{\text{total amount of TFV}}{\text{total amount of NFs}}\times 100\%. \end{array}$$

For NFP-loaded NFs, the samples were weighed and submerged in 5 mL of a chloroform/ethanol (3:1) mixture for 2 h to allow the complete dissolution of the PCL fiber. The solution was vortexed for 5 min and analyzed using a UV spectrophotometer at 240 nm. Drug loading was the same as in equation (2).

The drug entrapment efficiency was calculated as the percentage difference between the initial drug concentration in the polymer-drug dispersion and the amount of drug content measured in the NF after evaluation.

### 2.8. *In Vitro* Degradation Analysis

Degradation studies were conducted to evaluate the responsiveness of electrospun fibers to variations in vaginal pH, specifically at pH 6.8, which reflects the average vaginal environment following semen release.

Square samples measuring 1.76 cm^2^ were excised from dried electrospun fibers. The samples were immersed in PBS (pH 6.8) at a controlled temperature of 37°C. Short-term immersion periods of 30 s and 2 min were selected to capture initial morphological alterations indicative of the fibers' integrity under physiological intravaginal conditions.

Following immersion, the samples were dried and subsequently analyzed using SEM to assess structural changes under degradation process conditions. The diameter and porosity of the fibers were measured and compared between the 30 s and 2 min intervals. This comparative analysis aimed to elucidate degradation kinetics and assess the morphological stability of the fibers in response to physiological conditions.

### 2.9. *In Vitro* Drug Release Analysis

NF pieces with a cross-sectional area of 4 cm^2^ were cut and placed in 20 mL borosilicate glass bottles on a thermostatically controlled magnetic stirrer. This procedure was performed in triplicate for both SVF and a combination of SVF and simulated seminal fluid (SSF).

Prewarmed (37°C) SVF (pH 4.2) and a ratio of SSF (pH 7.4) to SVF (3:1) were used to simulate normal vaginal pH (4–6) and postsemen entry pH (6.5). The bottles were maintained in a water bath set to 37 ± 1°C. Liquid samples (2 mL) were taken at 0.083, 0.25, 0.5, 1, 4, and 24 h. These samples were replaced with fresh media to maintain sink conditions.

Standard solutions of TFV and NFP were prepared at 100 μg/mL in SVF (25 mM, pH 4.3) and PBS (pH 7.4), and further diluted to create calibration curves. Quantification was performed using these standards, with eluate from blank fibers in SVF used as background correction. The released TFV or NFP was quantified by measuring absorbance at their respective peak maxima (260 nm for TFV and 240 nm for NFP) using a UV-vis spectrophotometer (UV-2005, J.P. Selecta, Spain). All measurements were performed in triplicate, and absorbance values were converted into TFV/NFP concentrations using a standard curve (*r*^2^ = 0.99).

The analytical methods were validated using standard solutions and spiked samples to confirm the absence of interference from blank polymer fibers. The drug release kinetics were observed under pH conditions relevant to vaginal drug delivery [15].

#### 2.9.1. Drug Release Kinetics

The drug release kinetics of the NFs were evaluated using several kinetic models, including the zero-order, first-order, Higuchi, and Korsmeyer–Peppas models. The selection of the most suitable model was determined by the coefficient of determination (*R*^2^) and the release exponent (*n*).

### 2.10. Sperm Motility Tests

Human semen samples were collected through masturbation from volunteer donors at an assisted conception unit, with ethical approval and informed consent. The samples were analyzed for semen volume, sperm concentration, and motility. Only samples with normal parameters, including a minimum sperm concentration of 20 × 10^6^ per milliliter, were selected for use. The samples were ejaculated into sterile containers and allowed to liquefy at room temperature for 30 min prior to processing.

To each 1 mL of a sperm sample, 10 μL of dissolved PCL–NFP fibers was added. This was then incubated at 37°C *in vitro*. A 10 μL aliquot of the sample was placed on a microscope slide and covered with a cover glass (18 mm × 18 mm). At least six microscopic fields were observed for analysis. The motility of each spermatozoon was classified according to the WHO Laboratory Manual as follows:

a. Rapid and linear progressive motility
b. Slow or sluggish linear or nonlinear movement
c. Nonprogressive motility
d. Immotile

Each slide was examined twice, and motility data were recorded at two time points: after 1 h and 2 h of incubation. Sperm motility inhibition was expressed as a percentage. The number of motile and nonmotile spermatozoa was counted initially at 0 h and then at 15-min intervals over the following two hours.

### 2.11. Safety Assessment Studies

The safety assessment study was conducted using a modified rabbit vaginal irritation (RVI) test model. Healthy female adult naïve rabbits (weighing 2–5 kg) were obtained from Nomad Farms, Ogun State, Nigeria. All animal tests were conducted in compliance with the NIH guidelines for the care and use of laboratory animals as well as institutional guidelines, with approval from the Health Research Ethical Committee (approval number: CMUL/ACUREC/11/23/1310). The results of the *in vivo* studies were presented according to the ARRIVE documentation standards.

The rabbits were kept in a temperature-controlled facility (22 ± 2°C and RH 40 ± 3%) with a 12 h light/dark cycle for at least one week before usage, with unrestricted access to food and water. Five days before the application of the electrospun scaffold, all rabbits received a subcutaneous injection of medroxyprogesterone acetate (2 mg/kg/body weight; Depo-Provera®, Pfizer, NY, USA) for hormonal synchronization. The rabbits were divided into four groups of three, and the formulation was administered intravaginally to each group once a day using a sterile steel needle with a ball-point end to minimize discomfort.

The exterior aspect of the vagina was studied daily for difficulties in inoculation, vaginal contraction, excessive animal cries, spasms, redness, and burning. On the third day, the rabbits were humanely euthanized by exposing them to carbon dioxide gas. Vaginal tissues were removed and placed in sterile sample vials with 10% formalin solution for tissue examination. The samples were analyzed in sections using a Leica DM 750 microscope and ICC HD 50 camera at ×100 magnification [15].

### 2.12. Statistical Analysis

The data obtained were statistically analyzed with GraphPad Prism software (Version 9.5.0) and checked for normality. Data that followed a normal distribution were presented as mean ± standard deviation. Statistical analysis was performed using one-way ANOVA with Tukey's multiple comparisons test, or an unpaired *t*-test with Welch's correction. A *p* value of less than 0.05 was deemed statistically significant and is denoted by either an asterisk (∗) or a lowercase letter.

## 3. Results and Discussion

### 3.1. Surface Morphology of Electrospun NF

**Figure 1:**
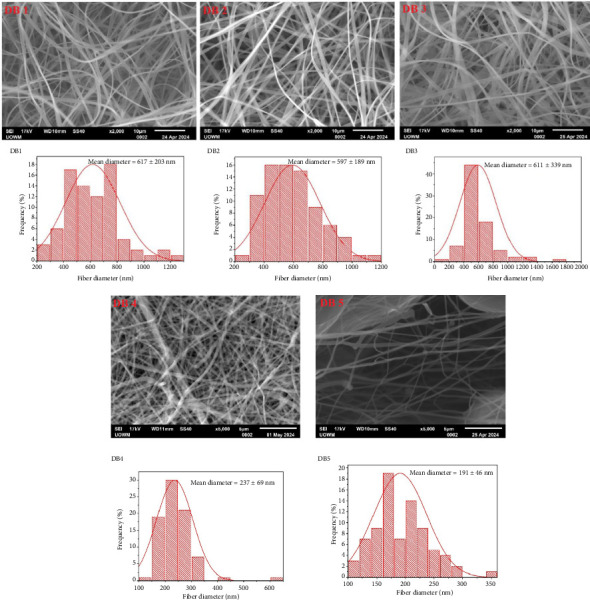
SEM images and fiber diameter distributions with average fiber diameters (AFDs) (*n* = 80) of DB 1–5 nanofibers.

Figure 1 depicts SEM images illustrating the surface morphology of all NFs. Except for DB5, all NFs display uniform and smooth surfaces without beads. DB1, DB2, and DB3 exhibit flat, ribbon-like fibers, likely due to the rapid evaporation of acetone, a highly volatile solvent used in their preparation, from the fibers [14]. The DB5 formulation exhibited considerable beading, which may be due to the use of crushed tablets rather than pure compound powder. Excipients and fillers present in tablet formulations can interfere with the homogeneity and viscosity of the spinning solution, potentially disrupting the electrospinning process and leading to bead formation instead of smooth, continuous fibers.

**Figure 2:**
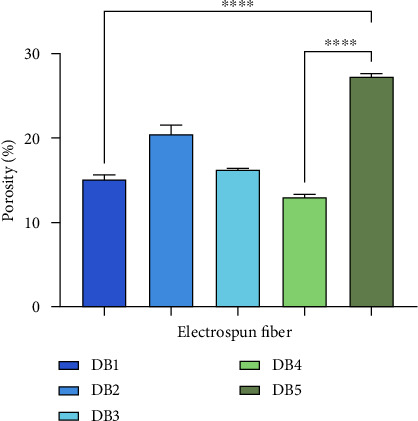
Porosity of DB1, DB2, DB3, DB4, and DB5 nanofibers. ^∗∗∗∗^Significant differences between samples ^∗∗∗∗^(*p* < 0.0001).

Introducing 10% w/w PEG reduces the average diameter by lowering viscosity, which enhances the stretching and elongation of the polymer solution [17]. The surface morphology of DB1–3 indicates consistent electrospinning parameters. However, adding TFV to the CAP/PEG blend slightly increases the NF diameter. The increased viscosity caused by TFV probably slowed the mass flow rate during spinning, contributing to thicker fiber formation. Porosity measurements indicate a general trend of increasing porosity with decreasing fiber diameter (Figure 2).

In contrast, DB5 shows significant bead formation, likely due to electrospinning jet instability. Beads can negatively impact drug loading and release kinetics [18]. DB4 exhibits a porosity of 15.88 ± 0.5%, while DB5 shows a porosity of 27.36 ± 0.27%. This increase is attributed to the presence of bead defects, which disrupt the uniform fiber structure and may negatively impact the mechanical integrity of the fiber.

### 3.2. Mechanical Characterization

**Figure 3:**
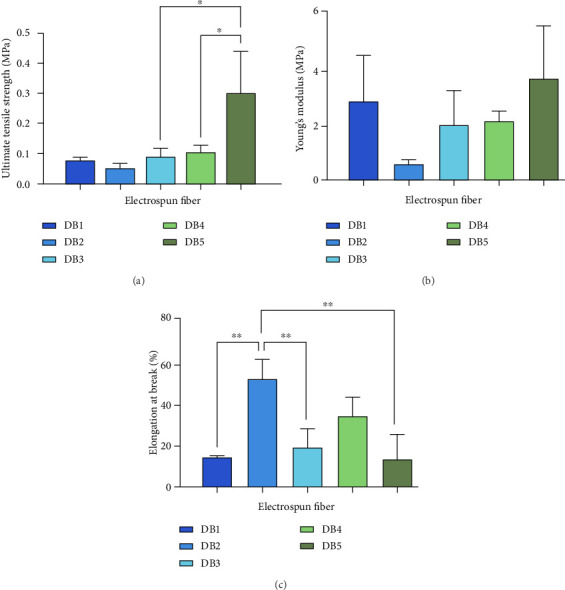
Ultimate tensile strength, Young's modulus, and elongation at break (%) for DB 1, DB 2, DB3, DB4, and DB5 nanofibers. ^∗^Significant differences between samples ^∗^(*p* < 0.05) and ^∗∗^(*p* < 0.01).

Introducing PEG into the 17% CAP solution reduced the ultimate tensile strength (UTS) of the fibers to 0.05135 ± 0.015 MPa, while the inclusion of TFV increased it to 0.09119 ± 0.025 MPa (Figure 3). Previous studies have indicated a correlation between fiber diameter and tensile strength in NFs. Nair et al., [19] observed this phenomenon in polyurethane-based electrospun fiber scaffolds, attributing lower strength in smaller diameter fibers to weaker junctions between interconnected, or potentially, defects within individual fibers. The inherently soft nature of CAP fibers results in low tensile strength and this was also observed by Hua et al. [20].

The inclusion of NFP in DB5 increased the UTS significantly from 0.1034 ± 0.023 to 0.2997 ± 0.140 MPa (Figure 3). This suggests that the incorporation of the drug increases fiber density, thereby enhancing the UTS.

The addition of PEG notably decreased Young's modulus to 0.6233 ± 0.1416 MPa, subsequently increasing to 2.062 ± 1.235 MPa upon TFV inclusion (Figure 3). The Young's modulus for DB4 was 2.205 ± 0.34 MPa, which significantly increased to 3.768 ± 1.917 MPa for DB5 upon the inclusion of NFP. Young's modulus reflects material stiffness, with higher values indicating greater resistance to deformation, as observed in DB1 and DB5. PEG functions as a plasticizer, reducing intermolecular interactions between polymer chains and thereby enhancing NF flexibility. Conversely, TFV incorporation is noted to counteract some of the plasticizing effects by limiting polymer chain mobility. NFP incorporation was also seen to increase the stiffness of the PCL NF.

Elongation at break (%) represents the percentage change in the length of a material and indicates its total pressure tolerance and ductility [21]. Incorporating PEG notably increased elongation to 53.17%, whereas TFV inclusion significantly reduced it to 19.61%. Li et al. [22] reported similar findings in PLA with 10%w/w PEG, showing increased elongation at break and decreased tensile strength. Thus, understanding the mechanical properties of the electrospun fibers is essential for optimizing their design and ensuring suitability for the intended application [23].

### 3.3. Chemical Characterization

#### 3.3.1. FTIR

**Figure 4:**
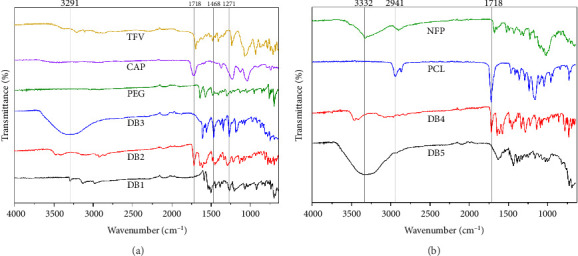
(a) FTIR spectra of CAP powder, PEG pellets, TFV powder, and DB1, DB2, and DB3 nanofibers. (b) FTIR spectra of PCL pellets and DB4 and DB5 nanofibers.

The FTIR analysis (Figure 4) provides insights into the molecular composition and structural characteristics of the DB3 and DB5 formulations. It is important to ensure that the fibers contain expected components and to check for any chemical interactions that may have occurred. In DB3, containing CAP, PEG, and TFV, characteristic peaks were observed for each component, indicating their presence and interactions within the formulation, consistent with previous reports [24, 25]. CAP exhibited peaks at 1318 and 1271 cm^−1^, corresponding to the C-O-C bonds in the phthalate ester groups. PEG introduced peaks at 3485 and 3410 cm^−1^, indicative of O-H stretching. TFV exhibited peaks at 1695 cm^−1^ (C=O stretching), 1069 cm^−1^ (C-O stretching), 1345 cm^−1^ (N-H bending), 1233 cm^−1^ (P=O linkage), and 1185 cm^−1^ (C-N stretching), highlighting its unique fingerprint, consistent with previous spectroscopic studies [26, 27]. A broad peak at 3302 cm^−1^ was attributed to intermolecular hydrogen bonding interactions between TFV, CAP, and PEG, similar to observations in other hydrogen-bonded systems [28]. The broad and strong peak of DB5 at 3332 cm^−1^ can be due to the N-H hydrogen bond interaction of NFP with PCL matrix [29]. The FTIR spectra of NFP showed a peak at 1681 cm^−1^ and a smaller peak at 1647 cm^−1^; these could be attributed to C=O and pyridine stretch similar to results obtained by da Costa et al., [30], which obtained 1688 and 1622 cm^−1^. The stretching vibration for the C-O-C stretch of PCL is observed at 1159 cm^−1^ in DB5 [31]. The peaks at 3462 cm^−1^ and 3414 cm^−1^ in DB4 signify asymmetric O-H stretching characteristic of PCL, while the sharp strong peak at 1718 cm^−1^ is attributable to C=O stretch for ester; multiple strong double peaks at 1647 and 1617 cm^−1^ and the peak at 1453 cm^−1^ are due to the stretching vibrations of the ester groups present in PCL [32].

#### 3.3.2. XRD

The XRD analysis revealed distinct crystalline patterns within the electrospun fiber formulations DB3 and DB5, influenced by their respective compositions and processing parameters. XRD analysis was used to determine the crystallinity of the biopolymers, as higher crystallinity is typically associated with improved mechanical strength in electrospun fibers [33]. DB5, containing 15% PCL loaded with the drug NFP, exhibited small peaks at 2θ values of 7.5° and 14.8° and a slightly higher peak at 27° in the XRD analysis. In comparison, pure PCL showed much larger and distinct peaks at approximately 2θ values of 20.1°, 21.6°, and 24.1°. This observation is consistent with the findings of other studies involving the encapsulation of drugs within PCL electrospun fibers. The result of the XRD analysis of the NFP sample confirmed its crystalline nature by major peaks at 2θ values of 8.3°, 12.7°, 14.8°, 16.5°, 20.1°, and 22.7° with the most intense peak at 20.1°. There were less prominent peaks at 37.9° and 44.1°. It is well-documented that NFP exists in a crystalline form, as reported by Jagdale et al., [34] when they observed distinct crystalline peaks for NFP at 2θ values of around 10.62°, 12.46°, 15.42°, 16.98°, 20.94°, and 22.80°. Alshaya et al. [35] also reported 2*θ* values of 8°, 15°, 19°, 21°, and 25° for NFP, which confirms the crystalline nature of the drug.

The small-sized peaks observed in DB5 formulation, containing 15% PCL loaded with the drug NFP, indicated that the crystallinity of PCL was nearly completely reduced upon successful encapsulation of NFP, resulting in DB5 exhibiting a more amorphous nature compared to the XRD spectra of pure NFP. Alshaya et al. (2022) reported the successful encapsulation of NFP in polyvinylpyrrolidone electrospun fibers, with the XRD patterns showing a significant reduction in the characteristic crystalline peaks of both polyvinylpyrrolidone and NFP, indicating their amorphous state within the fiber matrix. This amorphization can contribute to improved dissolution rates and bioavailability of poorly water-soluble drugs such as NFP.

**Figure 5:**
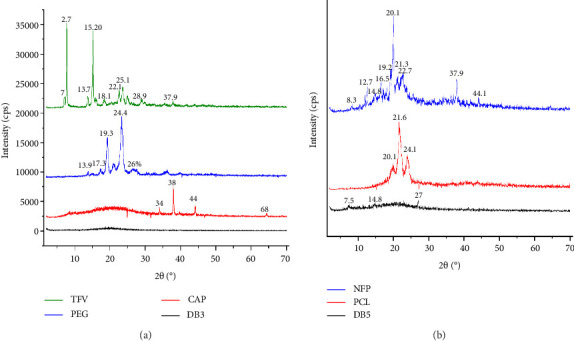
(a) XRD diffractograms of pure CAP, PEG, TFV powder, and DB3 nanofibers. (b) XRD diffractograms of PCL pellets and DB 5 nanofibers.

In contrast, DB3, comprising 17% CAP, 10% PEG, and 1% TFV, exhibited a predominantly amorphous profile without distinct peaks. The absence of pronounced crystalline features could be attributed to the complex composition and the presence of TFV, which may perturb polymer chain alignment and impede crystalline formations, aligning with prior studies exploring drug–polymer interactions and their impact on material morphology [36–38]. Upon encapsulation during electrospinning, TFV loses its crystallinity (Figure 5), indicating successful dispersion and encapsulation in the formulation.

For instance, Huang et al. [39] observed the loss of tenofovir crystallinity in the electrospun fibers, indicating successful encapsulation of the drug within the CAP polymer matrix. Similarly, Carson et al. [40] loaded TFV into PCL electrospun fibers for controlled release of the drug; they also reported an amorphous state of TFV within the PCL fibers.

The XRD patterns of both DB3 and DB5 exhibited a broad halo pattern characteristic of molecularly dispersed drugs, evident from the broad humps observed in the 2θ range of 11°–30° and 5°–30°, respectively. Qian et al., [37] found that various drug–polymer amorphous solid dispersions showed broad halos in their XRD patterns, indicating the amorphous nature of the drug dispersed in the polymer.

#### 3.3.3. DSC

**Figure 6:**
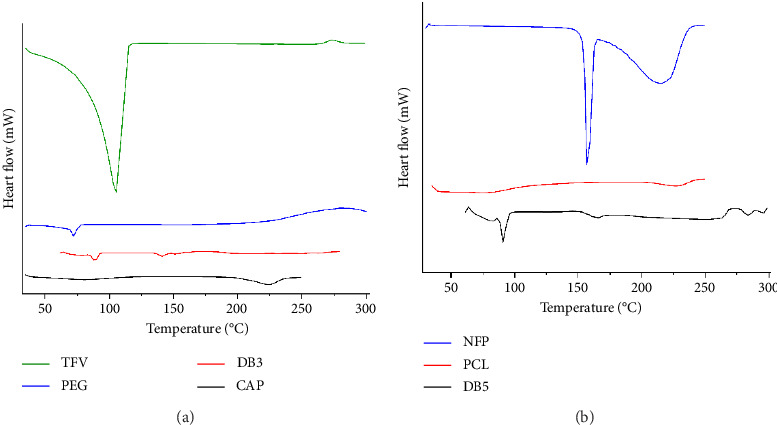
(a) DSC thermograms of pure CAP, PEG, TFV powder, and DB3 nanofibers. (b) DSC thermograms of pure CAP, PEG, TFV powder, and DB3 nanofibers.

DSC analysis characterized the thermal behavior of the formulations. For PCL, an endothermic trough at 73.94°C indicated melting, while a trough at 225.66°C suggested decomposition, consistent with the reported values [41, 42]. The DSC thermogram of NFP exhibited an endothermic peak at 156.83°C, indicating a distinct melting point and suggesting that the sample is pure and crystalline. This melting temperature implies that the NFP sample likely contained more of Polymorph III, one of the polymorphic forms of NFP, as reported by Riga et al., [43]. The second broad endothermic peak at 216°C indicates a more complex thermal event, possibly decomposition, occurring over a wider temperature range. This observation is consistent with values obtained in previous studies which indicated that the decomposition temperature of the NFP is within 210°C–390°C [44]. Sample DB5, comprising 15% PCL and NFP, exhibited a crystallization peak at 146.03°C and a potential melting trough at 90.75°C which was lower than the melting point of pure NFP (173°C) from literature, indicating the encapsulation of NFP within the polymer matrix [45]. The interactions between drugs and polymers may have resulted in the endothermic peak at 165.96°C [29]. Sample DB3, containing CAP, PEG, and TFV, displayed endothermic troughs at 78.28°C, 87.5°C, 141.24°C, and 150.65°C, indicating potential phase transitions or melting events, similar to reported values for CAP-based formulations [46, 47]. The strong endothermic trough at 87.5°C in the DSC thermogram of DB3 indicated melting as shown in Figure 6. Pure CAP exhibited endothermic troughs at 80.37°C and 223.09°C, suggesting melting and thermochemical transition related to acetyl bond breaking respectively [48]. An exothermic peak at 171.74°C for DB3 suggested crystallization, while subsequent troughs at 215.99°C, 232.32°C, and 250.99°C indicated further thermal transitions or decomposition, consistent with the complex nature of the formulation [28].

### 3.4. *In Vitro* Mucoadhesion Analysis

An enhanced mucoadhesion is crucial for prolonged drug release and retention along the vaginal mucosa, improving contact time and ensuring the complete release of TFV, thereby aiding in the inactivation of the virus within the vagina [49].

**Figure 7:**
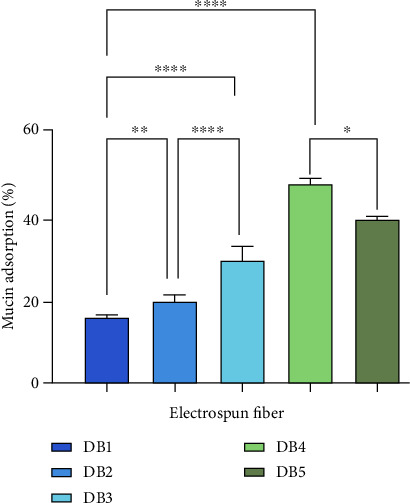
Mucin adsorption (%) of DB1, DB2, DB3, DB4, and DB 5 nanofibers. ^∗^Significant differences between samples ^∗^(*p* < 0.05), ^∗∗^(*p* < 0.01), and ^∗∗∗∗^(*p* < 0.0001).

The mucoadhesive interaction between the mucosal surface and the polymer is influenced by several physical and chemical factors. These include the polymer's molecular weight, plasticity, cross-linking, swelling behavior, spatial conformation, concentration, surface charge, availability of hydrogen bonding sites, and the pH of the mucoadhesive interface. [50]. The mucoadhesion study was carried out using the mucin from Porcine stomach mucin-type II, and the results are shown in Figure 7. Mucin adsorption increased significantly across the samples, with DB1, DB2, and DB3 exhibiting average adsorption values of 13.68 ± 0.24%, 19.34 ± 0.94%, and 29.01 ± 2.83%, respectively. The incorporation of PEG enhanced mucin adsorption significantly, by approximately 40%. PEG has been described as an adhesion promoter, facilitating the mucoadhesion process via interpenetration [51]. The increased mucoadhesion observed upon incorporation of TFV may be attributed to its hydrophilicity, which facilitates better interaction with mucin, in addition to other contributing factors. The blank DB4 fiber exhibited a mucoadhesion of 52.93 ± 0.70%, which was significantly reduced upon NFP loading in DB5. It can be observed that PCL exhibits significantly higher mucoadhesion compared to CAP. This characteristic can enhance the residence time and enable the sustained release of NFP over an extended period. As a backing layer for CAP, PCL can also extend the residence time of CAP, ensuring the complete release of TFV within the desired timeframe.

### 3.5. Drug Loading and Entrapment Efficiency

**Figure 8:**
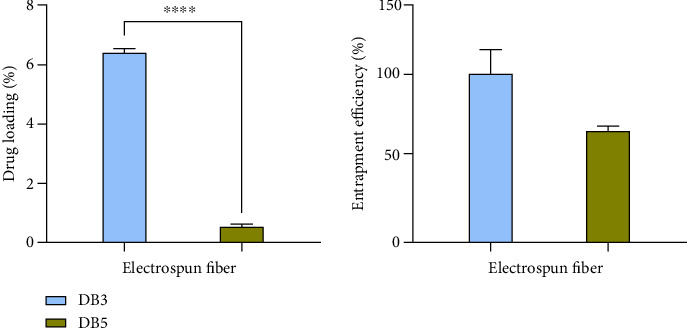
Drug loading (%) and entrapment efficiency (%) of DB 3 and DB 5 nanofibers.

DB3 exhibits a drug loading of 7.00 ± 0.27%, whereas DB5 shows a much lower drug loading of 7 0.56 ± 0.05%. The average weight of a 1.76 cm^2^ section was 3.93 ± 0.45 mg for DB3 and 5.40 ± 0.65 mg for DB5. The entrapment efficiencies are highlighted: DB3 with 52.13 ± 4.13% and DB5 with 63.86 ± 1.88% (Figure 8). These fibers fall short of achieving 100% E.E (%).

Several factors contribute to this less-than-perfect entrapment efficiency. One critical issue is the supersaturation of the polymer matrix, where the concentration of drug molecules exceeds their solubility limit. This can cause some drug molecules to precipitate out of the solution, preventing their incorporation into the NFs. Moreover, incomplete dissolution of drug molecules in the solvent solution can lead to uneven distribution and suboptimal encapsulation within the NFs.

During the electrospinning process of DB3, rapid evaporation of solvents at the needle tips presents another challenge. This quick evaporation of acetone resulted in the solidification of polymer material at the needle tip, requiring occasional manual removal. Also, TFV was not completely soluble in the solvent system resulting in some visible sedimentation within the syringe. TFV molecules trapped within this solidified polymer are consequently excluded from the final NF structure, further reducing overall entrapment efficiency.

### 3.6. *In Vitro* Degradation Analysis

**Figure 9:**
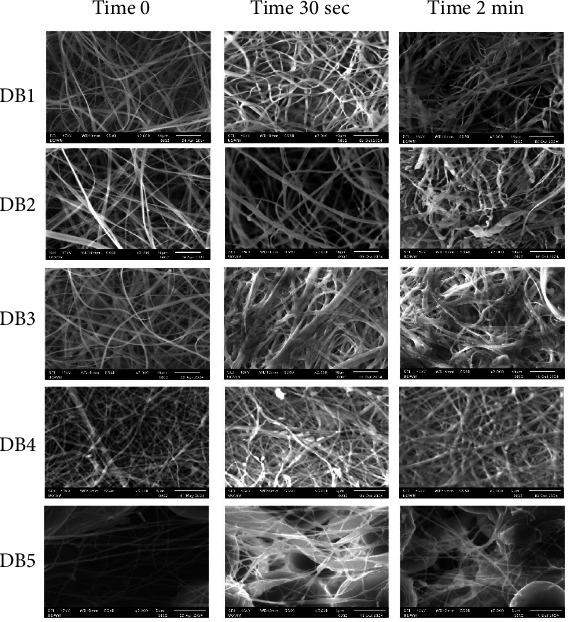
SEM images of DB1, DB2, DB3, DB4, and DB5 electrospun fiber samples after 30 s and 2 min of immersion in PBS (pH = 6.8).

Upon incubation in the degradation medium, fiber diameters in DB1, DB2, and DB3 increased after 30 s due to moisture absorption, as shown in Figure 9. This swelling is attributed to polymer chain relaxation within the matrix. After 2 min, a slight reduction in diameter was observed, likely due to matrix degradation and consequent loss of polymer material and structural integrity.

Despite the increase in fiber diameter, the average porosity increased across samples DB1, DB2, and DB3. This increase could be due to the initial swelling in the first 30 s, which expanded the spaces between fibers, and as degradation progressed, these spaces became more pronounced. In DB2 and DB3, which contain PEG as a plasticizer, fiber diameters increased by approximately 28% and 33%, respectively, after 30 s. In contrast, DB1, which did not contain PEG, showed only a 2.1% increase in diameter. This difference can be attributed to PEG's hydrophilic nature, which enhances its ability to absorb and retain moisture. The average porosity of DB2 and DB3 increased by only 4% and 12.5%, respectively, compared to DB1, which saw an increase of around 66%. This difference may be due to the stabilizing effect of PEG, which maintained fiber integrity by limiting the formation of voids within the fiber structure. SEM images of DB3 (at 2 min) also showed the deposition of TFV particles on the surface and within the degrading fibers.

In sample DB4, which contained PCL, the fiber diameter increased to 250 ± 93 nm after 30 s and to 270 ± 82 nm after 2 min. In DB5, which included NFP, the average fiber diameter initially increased by 29.3% to 247 ± 79 nm after 30 s but then reduced to 204 ± 50 nm after 2 min. The average porosity of DB4 increased by 16.5% after 30 s and decreased by 34.2% after 2 min. In DB5, the porosity reduced by 4.89% after 30 s and by a further 37.04% after 2 min. This change in porosity could be attributed to the increase in fiber diameter as the polymer initially absorbed the degradation medium, which closed the voids between the fibers.

PCL is a semicrystalline polymer, meaning it includes both amorphous and crystalline areas [52]. The gradual increase in average fiber diameter is likely attributable to the interaction between the degradation medium and the amorphous regions of the polymer, where moisture absorption occurs. It has been noted that thinner matrices with larger surface areas and increased pore sizes enhance water infiltration into the structure and the polymer matrix, while also promoting the diffusion of degradation products out of the matrix [53].

### 3.7. *In Vitro* Drug Release Kinetics

Drug release from NFs is a complex process influenced by factors such as matrix material composition, structure (including porosity and gel state), and environmental conditions such as pH, enzyme presence, and external triggers such as temperature [54]. *In vitro* diffusion profiling is crucial for predicting the behavior of drugs *in vivo* [55].

Mathematical modeling of drug release kinetics establishes a foundation for investigating the mass transport mechanisms responsible for regulating drug release. Typically, diffusion, erosion, and degradation represent the primary mechanisms governing the movement of solutes from polymeric matrices [56].

Certain polysaccharides, such as CAP, can swell or degrade in response to chemical stimuli such as pH, making them essential in controlled release systems. These systems aim to maintain optimal drug levels in the bloodstream or target tissues over time. Initially, achieving therapeutic drug concentrations swiftly through rapid release is crucial. Subsequently, precise drug release kinetics are necessary to sustain therapeutic doses over the desired duration [54].

PEG's solubility in both aqueous and organic solvents allows for incorporating hydrophobic drugs into matrices, as was performed to enhance the bioavailability of TFV in the vaginal environment.

**Figure 10:**
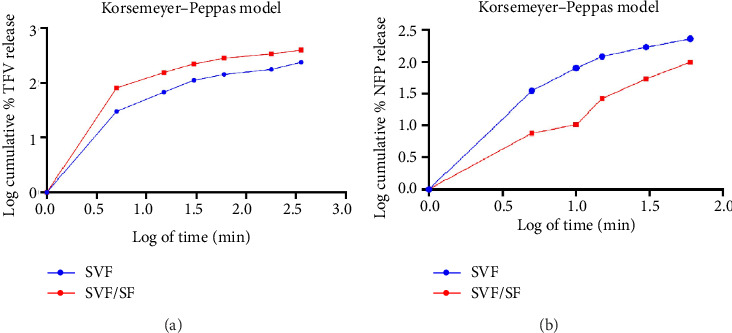
Korsmeyer–Peppas model showing the release profiles of (a) TFV from DB3 and (b) NFP from DB5, respectively.

Figure 10 illustrates the release profiles of TFV from CAP/PEG NFs loaded with 1% w/v TFV in SVF and SVF with SVF/SSF. TFV exhibited an initial burst release with concentrations of 151.6 μg/mL in SVF and 346.7 μg/mL in SVF/SSF at 0.25 h. At 5 h, TFV concentrations were 168.5 μg/mL in SVF and 268.5 μg/mL in SVF/SSF.

The *in vitro* drug release data were analyzed using various mathematical models to understand the release mechanism. In the SVF medium (Table 2), TFV's *in vitro* diffusion profiles from the NFs did not fit zero-order kinetics (*r*^2^ = 0.4710) or first-order kinetics (*r*^2^ = 0.3337) closely but closely adhered to Higuchi's equation (*r*^2^ = 0.9917), suggesting diffusion as the predominant release mechanism (Higuchi, 1961). For swellable systems such as electrospun fibers, Higuchi and Korsmeyer–Peppas models are commonly used due to their ability to account for swelling effects and non-Fickian diffusion behavior.

In the SVF/SSF medium (Table 3), TFV's *in vitro* diffusion profiles also did not closely fit zero-order kinetics (*r*^2^ = 0.0032) or first-order kinetics (*r*^2^ = 0.2282) but closely followed Higuchi's equation (*r*^2^ = 0.8895), indicating diffusion as the primary release mechanism [57]. The medium likely influences the relaxation of CAP polymer chains within the NF structure, facilitating drug diffusion until complete erosion occurs and releasing the entire drug payload [54].

Both in SVF and SVF/SSF, the Korsmeyer–Peppas model provided a good fit with diffusion exponents, *n* = 0.3491 and *n* = 0.2350, respectively, indicating Fickian diffusion and time-dependent release behavior. This model, tailored for polymeric matrices, elucidates the release mechanism and type through its exponent (*n*). An exponent of *n* ≤ 0.45 theoretically indicates Fickian diffusion, while values between 0.45 and 0.89 suggest mass transfer under non-Fickian diffusion [58], indicating Fickian diffusion in both media.

The initial rapid release observed is attributed to the dissolution of particles on or near the surface. Although the release data follow Higuchi kinetics, there is a noticeable trend of decreasing release rates as the solute amount diminishes within the matrix [57], indicating effective control of the release rate by the matrix.

Figure 10 illustrates the release profiles of NFP from PCL NFs loaded with 0.1% w/v NFP in SVF and SVF with SVF/SSF. In SVF, the initial release of NFP showed concentrations of 10.448 μg/mL at 0.25 h, increasing to 17.522 μg/mL by 5 h. On the other hand, SVF/SSF exhibited lower initial concentrations of 2.272 μg/mL at 0.25 h, reaching 10.448 μg/mL by 5 h.

Sailema-Palate et al., [59] summarized their research on PCL degradation under varying pH conditions and its implications for drug release kinetics. They found that PCL's degradation behavior varies with pH due to its impact on surface hydrophilicity. At alkaline pH (13), the polymer remains hydrophobic, which limits water absorption and restricts degradation to the surface. In contrast, under acidic conditions, PCL becomes more hydrophilic over time, allowing greater water uptake and promoting deeper hydrolytic degradation. PCL degrades differently under varying pH conditions due to changes in hydrophilicity. In acidic SVF (pH 4.2), PCL becomes more hydrophilic, increasing water uptake and accelerating hydrolytic degradation. This enhances porosity and surface area, leading to a pronounced initial burst release of NFP. In contrast, at pH 6.5 (SVF/SSF), PCL retains more of its hydrophobic character, slowing water penetration and degradation, which results in a more sustained drug release profile.

Mathematical modeling using Higuchi's equation and the Korsmeyer–Peppas model confirmed that diffusion is the primary mechanism governing NFP release from PCL NFs in both SVF and SVF/SSF. Higuchi's equation, which correlates drug release with the square root of time, highlighted the significant role of diffusion through the PCL matrix. The Korsmeyer–Peppas model provided a good fit with diffusion exponents (*n* = 0.4356 for SVF and *n* = 0.9619 for SVF/SSF), indicating Fickian diffusion in SVF and the higher exponent in SVF/SSF suggests a release mechanism closer to zero-order, implying complex interactions between polymer relaxation and drug diffusion influenced by pH conditions.

### 3.8. Sperm Motility Tests

Of the growing array of targets available in the field, the voltage-gated calcium ion channel CatSper, found exclusively in sperm and functionally relevant only in mature sperm, stands out as a highly promising target for male contraception [60]. When activated, CatSper triggers a significant calcium influx into the sperm tail, generating calcium oscillations that travel towards the head [61]. This intracellular calcium increase is crucial for processes such as the acrosome reaction, capacitation, and hyperactivated motility (HAM). HAM involves a shift in sperm flagellar beat patterns, facilitating detachment from the oviduct wall, efficient swimming in viscous environments, and penetration of the zona pellucida, which are essential for fertilization [62].

We investigated the spermicidal potential of electrospun fibers loaded with NFP in line with existing literature suggesting that NFP disrupts sperm motility by blocking calcium channels crucial for hyperactivation and the acrosome reaction [62-65], both essential for sperm function.

**Figure 11:**
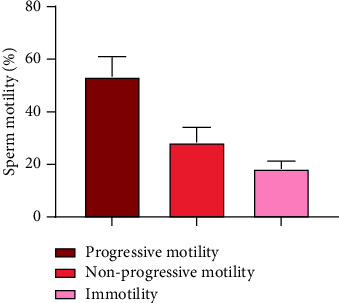
Effect of dissolved DB5 nanofibers (from 1.76 cm^2^ cross-sectional area) on sperm motility.

We investigated the effect of the NFP dissolution solution obtained from a cross-sectional area of 1.76 cm^2^ of NFP-loaded fibers on sperm motility. This was prepared by dissolving the fibers in PBS (pH 7.4). NFP fiber solution showed a progressive motility of 53.33 ± 7.63% (Figure 11). This suggests that the observed effect showed an alteration in sperm motility; however, its influence on HAM was not reflected in standard sperm motility tests conducted in this study. NFP acts as a CatSper channel blocker albeit not selectively, primarily targeting the hyperactivation of sperm cells, a process directly linked to CatSper activation.

### 3.9. Safety Assessment Studies

**Figure 12:**
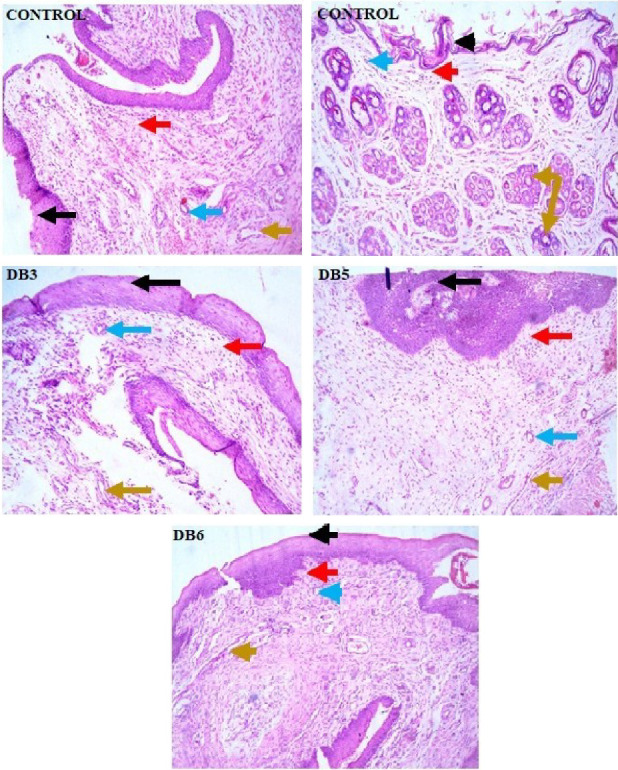
Histological images stained with hematoxylin and eosin were captured at an objective magnification of ×40, showing the control and DB 3, DB5, and DB6. ^∗^Black arrow: loss of the outer layer of epithelial exfoliation. Red arrow: lamina propria. Blue arrow: blood vessels. Yellow arrow: vascular congestion. Brown arrow: normal gland. Green arrow: inflammatory cells (H&E stain. Scale bar: 100 µ).

The safety assessment tested for signs of vaginal irritation from the administration of the NF samples. Histopathological examination of vaginal tissues (Figure 12) indicated no alterations in normal vaginal histology in rats after 7 days of fiber administration. In the control group, the tissue appeared healthy, characterized by a well-structured, elastic fibrovascular stroma, indicative of normal vaginal tissue. The histological section of the outer vaginal tissue of DB3 demonstrated complete exfoliation of the outer layer of stratified squamous epithelium, leaving the basal cell layer, which is typical of tissues undergoing normal cell turnover. The lamina propria appeared as a thick fibrovascular stroma with elastic fibers, and no inflammatory cells or signs of apoptosis or congested blood vessels were observed, indicating no immune response or pathological inflammation. The histological section of the outer vagina tissue treated with DB5 shows no indications of inflammation, apoptosis (cell death), or congested blood vessels. These findings emphasize the healthy structural and physiological condition of vaginal tissue, demonstrating the absence of inflammation or apoptosis, the presence of a vascular supply, and the functional role of glands in supporting tissue health. In their study, Machado et al., [63] found that TFV films and TFV-PLGA/SA NPs-in-film did not cause adverse effects on general health, behavior, weight, or external genitalia in mice. Histological analysis showed no significant changes, suggesting potential protective effects of the film formulation.

## 4. Conclusion

In this study, we developed and characterized electrospun MPT NFs to demonstrate their potential for effectively protecting against HIV-1 infection while also providing contraception. The NFs exhibited favorable biopharmaceutical properties, including excellent drug loading capacity, absence of toxicity to the tissues of the female genital tract, and pH-responsive drug release patterns. TFV demonstrates on-demand release with a small initial payload prior to a pH change, while NFP exhibits sustained drug release. This dual-release mechanism could potentially provide a broad protection window against HIV-1 infection and unplanned pregnancy. The study examined the impact of NFP-loaded fibers on sperm motility, revealing a reduction in progressive motility compared to the control. This engineered multilayered electrospun fibers MPT has demonstrated promising safety and efficacy as well as a platform for delivery of other APIs, underscoring the potential of this innovative drug delivery system to provide dual protection against HIV-1 infection and unplanned pregnancy.

## Figures and Tables

**Table 1:** Formulation design for the polymer mixtures.

Electrospun fiber	CAP (%w/v)	PEG (%w/w)	TFV (%w/v)	PCL (%w/v)	NFP (%w/v)
DB1	17	—	—	—	—
DB2	17	10	—	—	—
DB3	17	10	1	—	—
DB4	—	—	—	15	—
DB5	—	—	—	15	0.1
DB6	17	10	1	15	0.1

**Table 2:** *In vitro* drug release kinetics' study for TFV (from DB3) and NFP (from DB4) in SVF.

Formulation	Zero-order		First-order		Higuchi		Korsmeyer–Peppas	
	*r* ^2^	*k*_0_	*r* ^2^	*k*_1_	*r* ^2^	*k*_2_	*r* ^2^	*N*
DB3	0.4710	0.1217	0.3337	0.0048	0.9917	12.745	0.9972	0.3491
DB4	0.4627	0.0991	0.4397	0.0262	0.9745	12.301	0.9968	0.4356

**Table 3:** *In vitro* drug release kinetics' study for TFV (from DB3) and NFP (from DB4) in SVF/SF.

Formulation	Zero-order		First-order		Higuchi		Korsmeyer–Peppas	
	*r* ^2^	*k*_0_	*r* ^2^	*k*_1_	*r* ^2^	*k*_2_	*r* ^2^	*N*
DB3	0.0032	0.0105	0.2282	0.0033	0.8895	17.545	0.9990	0.2350
DB4	0.9498	0.1194	0.6530	0.0041	0.9548	5.3297	0.9879	0.9619

## Data Availability

Data will be made available on request.
